# Prescribed fires, smoke exposure, and hospital utilization among heart failure patients

**DOI:** 10.1186/s12940-023-01032-4

**Published:** 2023-12-13

**Authors:** Henry Raab, Joshua Moyer, Sadia Afrin, Fernando Garcia-Menendez, Cavin K. Ward-Caviness

**Affiliations:** 1 Center for Public Health and Environmental Assessment, US Environmental Protection Agency, Human Studies Building, 104 Mason Farm Rd, Chapel Hill, NC 27514 USA; 2 Department of Civil, Construction, and Environmental Engineering, North Carolina State University, Raleigh, NC 27606 USA; 3 Present address: MIT Laboratory for Aviation and the Environment, Massachusetts Institute of Technology, Cambridge, MA 02139 USA

**Keywords:** Prescribed fires, Smoke exposure, Hospitalizations, Heart failure

## Abstract

**Background:**

Prescribed fires often have ecological benefits, but their environmental health risks have been infrequently studied. We investigated associations between residing near a prescribed fire, wildfire smoke exposure, and heart failure (HF) patients’ hospital utilization.

**Methods:**

We used electronic health records from January 2014 to December 2016 in a North Carolina hospital-based cohort to determine HF diagnoses, primary residence, and hospital utilization. Using a cross-sectional study design, we associated the prescribed fire occurrences within 1, 2, and 5 km of the patients’ primary residence with the number of hospital visits and 7- and 30-day readmissions. To compare prescribed fire associations with those observed for wildfire smoke, we also associated zip code-level smoke density data designed to capture wildfire smoke emissions with hospital utilization amongst HF patients. Quasi-Poisson regression models were used for the number of hospital visits, while zero-inflated Poisson regression models were used for readmissions. All models were adjusted for age, sex, race, and neighborhood socioeconomic status and included an offset for follow-up time. The results are the percent change and the 95% confidence interval (CI).

**Results:**

Associations between prescribed fire occurrences and hospital visits were generally null, with the few associations observed being with prescribed fires within 5 and 2 km of the primary residence in the negative direction but not the more restrictive 1 km radius. However, exposure to medium or heavy smoke (primarily from wildfires) at the zip code level was associated with both 7-day (8.5% increase; 95% CI = 1.5%, 16.0%) and 30-day readmissions (5.4%; 95% CI = 2.3%, 8.5%), and to a lesser degree, hospital visits (1.5%; 95% CI: 0.0%, 3.0%) matching previous studies.

**Conclusions:**

Area-level smoke exposure driven by wildfires is positively associated with hospital utilization but not proximity to prescribed fires.

**Supplementary Information:**

The online version contains supplementary material available at 10.1186/s12940-023-01032-4.

## Background

Climate change is projected to increase wildfire occurrence and severity [1–3]. One consequence of increased wildfire activity is a decline in air quality, primarily from smoke. Wildfire smoke is a complex mixture with fine particulate matter (PM_2.5_) as a primary pollutant [4–6], and exposure to PM_2.5_ is associated with increased morbidity and mortality [7–9]. Conversely, reducing air pollution, and PM_2.5_ specifically, can lower morbidity and mortality, particularly related to cardiovascular disease [10–15]. Critical reviews of the health effects of wildfire smoke exposure provided evidence of consistent positive associations between wildfire smoke exposure and respiratory morbidity and all-cause mortality [2, 16].

Prescribed fires can be used to try and reduce the size, frequency, and severity of wildfires [17–19], a practice that is increasing [20]. Prescribed fires may also have ecological benefits like natural hazards regulation, pollination, and pest and disease control [21] that can motivate their usage in communities. However, prescribed fires also release smoke into the atmosphere, though in much smaller quantities than wildfires, primarily due to their smaller size [22]. Despite the smaller size of prescribed fires than wildfires, a study of two states in the southeastern United States (Florida and Georgia) found that prescribed fires were a primary source of air pollution and explained up to 50% of the variability in daily PM _2.5_ concentrations [23]. Prescribed fires may also have a disproportionate impact on socially vulnerable populations in the United States [24, 25], presenting a possible environmental justice issue. Although health effects from prescribed fire smoke are potentially a concern for vulnerable populations, there is a lack of studies investigating the associations between health effects and repeated prescribed fire exposures in clinically vulnerable individuals.

Heart failure is one of the most severe cardiovascular diseases. An estimated 6.0 million individuals in the US ≥ 20 years old have heart failure, which is projected to increase to 8.0 million individuals by 2030, partially due to an aging population [26, 27]. People with heart failure are at higher risk of health complications associated with air pollution than the general population [28, 29]. Previous studies have, in particular, shown elevated risks of readmissions and hospital visits among HF patients exposed to elevated concentrations of PM_2.5_ [30] and ozone [31]. In a study in the southeastern United States, associations between air pollution and hospitalization among HF patients were stronger than those with other cardiovascular diseases, highlighting the unique vulnerability of HF patients to air pollution exposure [31].

This study aims to evaluate the health effects associated with prescribed fire occurrences and smoke exposure from wildfires among heart failure (HF) patients. We used the Environmental Protection Agency Clinical and Archived Records Research for Environmental Studies (EPA CARES) for this study.

## Methods

### Study cohort

EPA CARES is an electronic health record (EHR) resource that merges EHRs from the University of North Carolina Healthcare System with environmental exposures [30, 32, 33]. The EPA CARES resource has previously been used to study HF patients' environmental health risks [30, 32, 33] but has not been used before to examine the potential impacts of proximity to prescribed fires or exposure to wildfire smoke. HF patients in the EPA CARES resource had a recorded HF diagnosis at a hospital or clinic affiliated with the University of North Carolina Healthcare System (UNCHCS) between July 1, 2004, and December 31, 2016. The study cohort for this analysis was restricted to patient observations recorded between January 1, 2014, and December 31, 2016, as the prescribed fire data covered only this period. As in previous analyses, HF was defined according to the International Classification of Diseases, Ninth Revision (ICD-9) codes 428.x and the International Classification of Diseases, Tenth Revision (ICD-10) codes I50.x [32] based on diagnoses recorded in the electronic health record. Individuals were then linked to demographics, address history, hospital and state death records, and hospital visits data (including inpatient, outpatient, and emergency room visits) as recorded in their EHR. We focused on hospital utilization independent of cause as this is the broadest possible capture of hospital visits, admissions, and readmissions. While the vast majority of these will be for HF, given the severity of the disease, air pollution exposure has broad impacts on multiple organ systems and thus may contribute to a variety of hospitalizations – including those outside of the commonly studied cardiovascular and pulmonary domains. Additionally, the EHR data did not specifically detail primary and/or secondary reasons for visits which can make it difficult to conclusively determine a reason, particularly in a patient population with a high prevalence of co-morbidities such as HF patients. Smoking status was missing from 6.3% of the cohort, thus it was not included in the models. However, a sensitivity analysis was performed where smoking status was imputed using multiple imputation chained equations as implemented in the *mice* package in R as done in previous analyses of this patient data [30, 33]. Addresses were considered successfully geocoded at the zip code level, and 99.9% of addresses met this criterion [32]. As with previous studies using the EPA CARES resource, we restricted the study to participants who resided in North Carolina.

### Prescribed fire data

Prescribed fire locations and areas used in this study are based on U.S. EPA’s 2014–2016 version 7 air emissions modeling platforms (2014v7.1 [34], 2015v7.1 [35], and 2016v7.2 [36]). The platforms’ fire data was derived from fire emissions inventory tools and national, state, and tribal agencies databases, including the National Oceanic and Atmospheric Administration’s Hazard Mapping System, the Monitoring Trends in Burn Severity (MTBS) fire products, and fire data compiled by the North Carolina Department of Environmental Quality. The Satellite Mapping Automated Reanalysis Tool for Fire Incident Reconciliation version 2 (SMARTFIRE2) was used to reconcile these multiple sources of space-borne and ground-based fire information into daily geolocated fires and areas burned.

Prescribed fires have been shown to generate elevated air pollution (PM_2.5_) concentrations up to 9 km from their location, with the highest concentrations occurring within ~ 500m [37], and the impacted area around a prescribed fire can vary based on location and other conditions [25]. The prescribed fire data for this study did not include estimates of PM_2.5_ generated, thus based on previous studies, we linked participants with all prescribed fires that occurred within 5 km of their primary residence as determined by their EHRs. Address changes were dated, allowing us to follow individuals over time even when they changed residences. For our exposures, we examined the number of prescribed fire occurrences within 5 km, 2 km, and 1 km of the patients’ primary residence as this was likely to capture both local (1 & 2 km) and broad area-level (5 km) effects of prescribed fires. While some studies have noted that elevated PM_2.5_ can be detected beyond 5km of a prescribed fire, this would only be associated with larger, less frequent prescribed fires which are also less likely to occur near populated areas. Given the size of most prescribed fires, we considered a 5 km cutoff a reasonable maximum distance for this analysis. As stated before, our exposure metric for prescribed fires was the number of prescribed fires within each radius, as we did not have access to measured or modeled estimates of air pollution from the prescribed fires. Thus, while our exposure is a proxy for air pollution due to the prescribed fire, it may also capture the ecological effects of prescribed fires that currently have unknown health effects, if any.

### Smoke density

We also examined smoke density data (which is primarily generated by wildfires) separate from the prescribed fire data. We did this to compare health effects from residing near the occurrence of prescribed fires (which has not been evaluated for associations with hospital visits or readmissions) to those from wildfire smoke exposure which has been repeatedly associated with hospitalizations [38–40]. Daily smoke density data from January 2014 to December 2016 were acquired from the National Oceanic and Atmospheric Administration’s Hazard Mapping System (NOAA HMS) [41], which was designed to capture wildfire smoke. Smoke exposure days were assigned to individuals based on their zip code tabulation area (ZCTA) of residence. Population centroids within the US Census ZCTAs were intersected with HMS data to obtain smoke densities at the ZCTA level. HMS smoke density is derived from a combination of observations from the Geostationary Operational Environmental Satellites (GOES) and polar satellites. The HMS smoke product (HMS Smoke) combines data from satellite observations and NOAA expert image analysts to define potential light, medium, and heavy smoke plumes, representing appropriate smoke concentrations between 0–10, 10–21, and 21–32 µg/m^3^ respectively. HMS smoke density data is designed to detect large area smoke from wildfires and not prescribed fire smoke, although some prescribed fire smoke may be captured within the data.

### Statistical analysis

We used a cross-sectional study design to assess repeated exposure to common events which have been understudied for prescribed fires and wildfire smoke exposure – as opposed to the short-term impacts as might be done using a case-crossover approach. We did not have enough data for a longer-term prospective analysis. The outcomes considered were total hospital visits and 7-day and 30-day readmissions, with x-day readmissions defined as an inpatient admission occurring within x days of discharge from a prior inpatient hospitalization identical to previous definitions used [30, 33]. Total hospital visits included outpatient as well as inpatient and emergency room visits, and thus broadly capture hospital utilization which follows previous publications [30, 33]. Associations with hospital visits and readmissions were modeled using quasi-Poisson regression (hospital visits) and zero-inflated Poisson regression (readmissions) models.

We utilized an identical confounder adjustment for total hospital visits and readmissions. We used demographic data from the hospital records and socioeconomic data from the 2010 US Census Data to adjust for age at HF diagnosis, sex, race, and the following 2010 census block group variables: percent urban, percent of households below the federal poverty line, percent of individuals with a high school education or more, percent unemployed, median household value, and percent of individuals on public assistance. Confounders were chosen a priori based on previous analysis of air quality and hospitalizations examining this patient cohort [30, 33]. To improve convergence, all continuous confounders were standardized to have a zero mean and a standard deviation of one. Socioeconomic data were taken from the US census as this data, e.g., income or poverty status, is not recorded within the electronic health record. While insurance status is occasionally extracted from EHRs as a proxy for individual-level socioeconomic status, the older age of our cohort meant that most individuals used Medicare as their primary insurer, substantially decreasing insurance as a proxy for socioeconomic status in this data. All models included an offset of log-transformed follow-up time which accounted for not all study participants being in the study for the entire 3-year period (some study participants were diagnosed with HF after Jan 1, 2014, and others died before the study ended).

Only a minority of patients have a readmission causing an excess of 0s in the readmission distribution compared to what would be expected in a Poisson distribution. Thus, as stated before, we utilized a zero-inflated Poisson model, as implemented in the *pscl* R package [42, 43], to model the outcome. The confounder adjustment for the zero-inflated Poisson model remained the same as detailed above (including the log follow-up time offset), and the excess zeroes were modeled using an intercept-only model identical to the modeling approach taken in previous analyses of readmissions in this cohort [30]. As previously mentioned, our prescribed fire exposures were the number of prescribed fire occurrences within 1 km, 2 km, and 5 km of a patient’s primary address. For smoke density data, exposures were the number of total smoke days and the number of light, medium, heavy, and medium or heavy smoke days to capture smoke concentration-dependent health effects. We combined medium and heavy smoke days, given the relative rarity of each of them. Each exposure was evaluated in a separate model. We removed patients with Tukey outliers for total visits using the interquartile range (IQR) score (third quartile + 1.5*IQR) as done for previous analyses [32]. There were no lower limit Tukey outliers as that value was negative, and one cannot have negative visits or readmissions. Also, as done in previous analyses of this cohort, we removed outliers for 7- and 30-day readmissions by examining the distribution of the readmissions and decided to consider 7-day readmission observations > 4 and 30-day readmission observations > 7 as outliers (Figs. S-1 and S-2).

We conducted several sensitivity analyses for this study. To examine sensitivity to geocoding precision, we ran analyses restricted to individuals with street-level geocoding. We examined associations after restricting to individuals with age ≤ 100, limiting age recording errors. We imputed missing smoking status data using multiple-imputation chained equations implemented in the *mice* package in R and pooled the five imputations to obtain an overall estimate [44]. For the imputation analyses, we performed five imputations of the data and then pooled the imputations and used the confounder adjustment described previously. We also examined associations without outliers removed to examine their influence on the observed associations. We also evaluated PM_2.5_ on the day of the prescribed fire as a secondary outcome using the same confounder adjustment model as before and using daily PM_2.5_ models validated for the study area and utilized in previous analyses of this study population [30, 33]. Finally, we examined associations after restricting to patients with only one recorded address over the study to limit potential exposure misclassification related to errors such as incorrect recording of dates of address changes. Results from all analyses are presented as the percent change in the number of hospital admissions or readmissions and associated 95% confidence interval (CI).

## Results

The study population was comprised of 8,495 participants. Participants were aged 20–116 at the time of their HF diagnosis (mean age 70.9 ± 14.2 years). Table 1 shows the clinical covariates for these study participants. The study population was primarily white (63.5%) and roughly equivalent for sex (female 51%). Over 60% lived in urban areas, while 70.5% were within 5 km of at least one prescribed fire, 20.2% were within 2 km, and 5.5% were within 1 km of a prescribed fire.

**Table 1:** Study cohort description including prescribed fire occurrence, smoke exposure, total visits, and readmissions for study participants

**Covariates,** ***N* = 8,495**	**Mean**	**SD**	**IQR**
Age (y)	70.9	14.2	62.0—82.0
Urbanicity (%)	61.9	42	10.1—100.0
Poverty (%)	17.3	14.6	6.5—24.4
High school or more education (%)	85	11.6	78.2 – 94.2
Unemployed (%)	10.2	7.5	5.1 – 13.6
Median household value ($)	185 972	106 814	109 900 – 230 600
Public assistance (%)	1.9	3	0.0 – 2.8
	**N**	**%**	
White	5398	63.5	
Black	2420	28.5	
Other	677	8	
Male	4166	49	
Female	4329	51	
Within 1 km of a prescribed fire	469	5.5	
Within 2 km of a prescribed fire	1715	20.2	
Within 5 km of a prescribed fire	5992	70.5	
**Prescribed Fire and Smoke Day Statistics 2014–2016**			
	Median	Mean	Max
Prescribed fires within 1 km	0	0.06	5
Prescribed fires within 2 km	0	0.3	7
Prescribed fires within 5 km	1	1.9	13
Light smoke days	32	36.2	168
Medium smoke days	2	2.4	14
Heavy smoke days	1	1.2	10
Medium + heavy smoke days	3	3.6	20
Total smoke days	35	37.8	188
**Hospital Visits and Readmissions Statistics**			
	Median	Mean	Max
Total hospital visits	5	8.3	37
7-day readmissions	0	0.1	4
30-day readmissions	0	0.4	7

Units for measurement for continuous variables are in parentheses

*SD* Standard deviation, *IQR* Interquartile range

### Geographic distribution of prescribed fires and smoke density

**Fig. 1:**
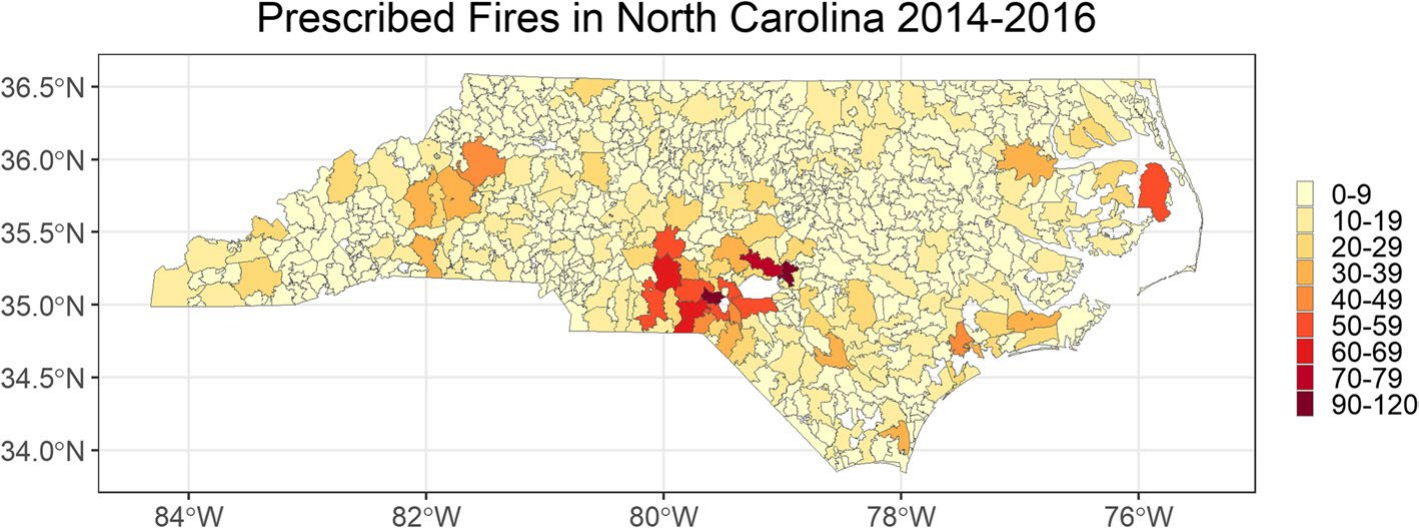
Prescribed fires in North Carolina between 2014–2016. Map shows the number of prescribed fires in North Carolina between 2014–2016 by ZCTA. Fires outside any ZCTA were assigned to the nearest ZCTA code. Due to the large number of prescribed fires in the Fort Bragg military base (776, 13.2%) they were excluded from this figure

Most prescribed fires in North Carolina are conducted in the south-central area of the state, which coincides with the presence of large military bases, as well as in the western and eastern regions (Fig. 1).

**Fig. 2:**
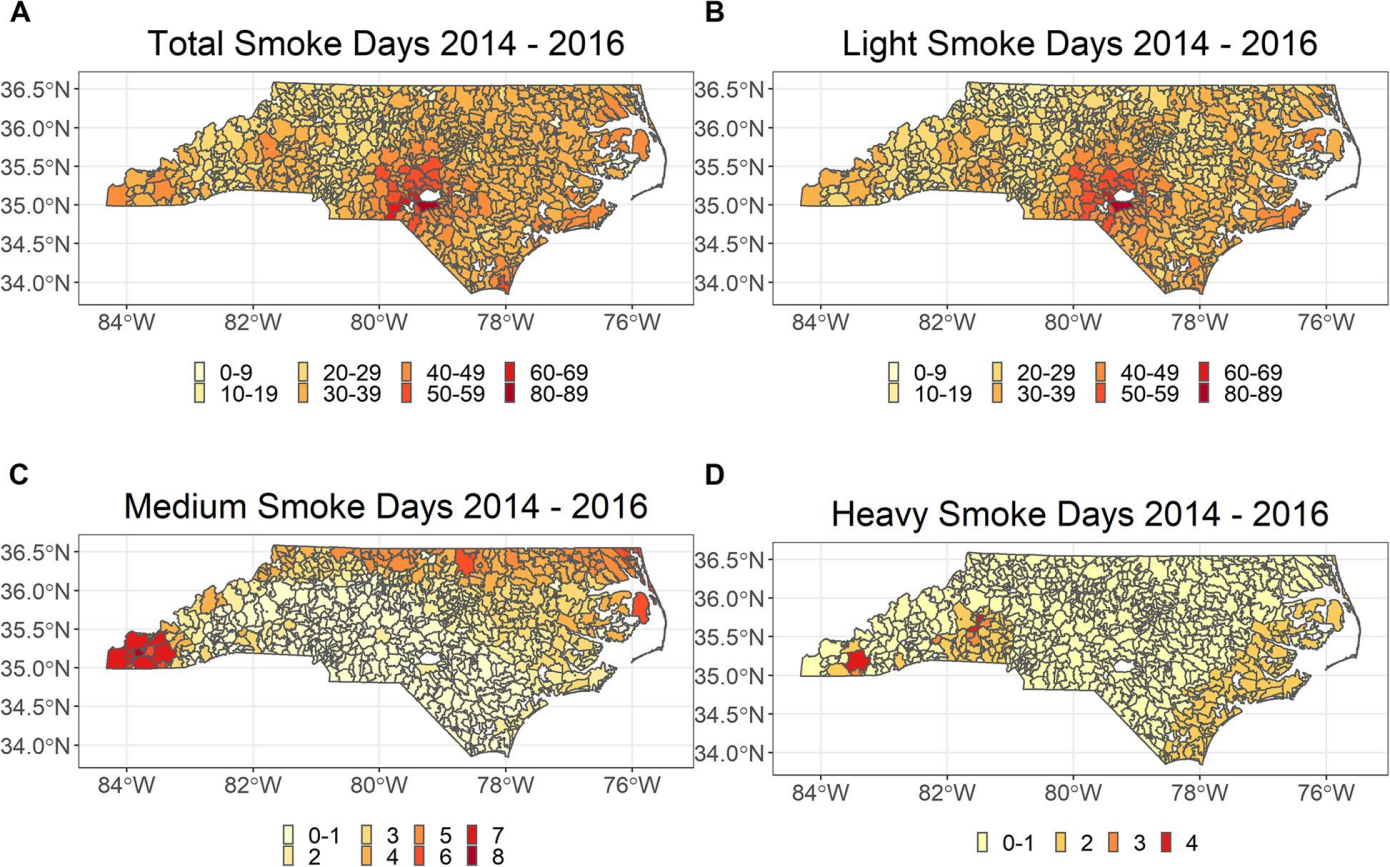
Number of smoke days in North Carolina between 2014–2016. Maps show total, low, medium, and heavy smoke days based on the HMS Smoke product between 2014–2016 by ZIP code tabulation area (ZCTA)

Military bases perform prescribed fires for several reasons, including reducing the risk of wildfires caused by artillery and range training [45, 46]. While a high number of fires are concentrated in a few distinct ZCTAs, the number of smoke days across the state based on smoke data from HMS varies significantly (Fig. 2).

Most light smoke days occurred in the south-central part of North Carolina and the state’s western and eastern regions (Fig. 2). The spatial distribution of total smoke days is similar to light smoke days since 90% of smoke days were from light smoke. Medium smoke days were primarily distributed in the northern and western regions of the state, while the heaviest smoke days occurred in the southeast and west. In the summer of 2016, drought conditions led to a very active fall fire season in western North Carolina and neighboring states [47]. These wildfires likely account for the high number of medium and heavy smoke days in western ZCTAs. The smoke data from HMS revealed 23,243 zip-smoke days between 2014–2016 in North Carolina.

**Fig. 3:**
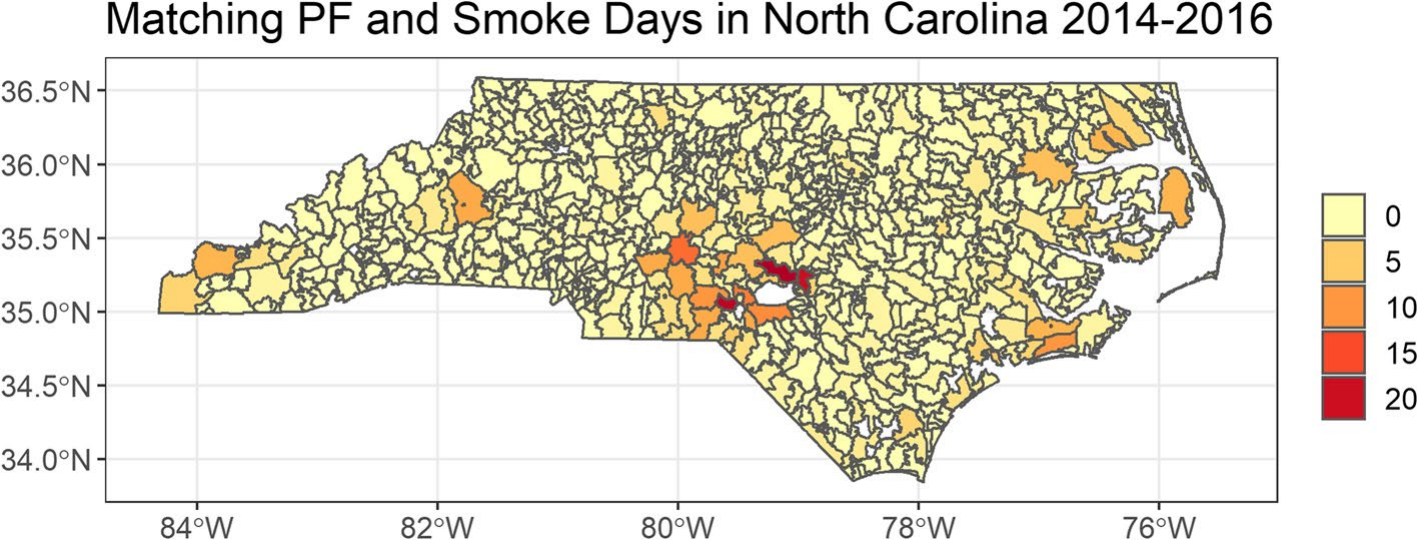
Matching prescribed fire and smoke days in NC 2014–2016*.* Map of the number of prescribed fires in each ZCTA the same day as a smoke day recorded by the HMS product

The total number of prescribed fires recorded in the state during this period was 5,840. Only 575 prescribed fires (9.8%) occurred on the same date and zip code as a smoke day (light, medium, or heavy) recorded in HMS (Fig. 3). This suggests that prescribed fires rarely (if ever) generate sufficient smoke to be captured by HMS, as the low frequency of co-occurrence could be random chance. The lack of intersection between smoke days recorded in HMS and prescribed fire occurrence reflects both the design of HMS (which uses satellites with a resolution ranging from 375m to 2km) and the small size of prescribed fires (median acres burned = 34).

### Associations with prescribed fires

For prescribed fire occurrences, we observed inverse associations with total hospital visits for an additional prescribed fire within 5 km (2.6% decrease per additional prescribed fire; 95% CI = -4.1%, -1.1%) and within 2 km (6.0% decrease per additional prescribed fire; 95% CI = -10.4%, -1.6; Table 2). For prescribed fires within 1 km, the association was still negative in direction but with a wide confidence interval fully encompassing the null. There was no association between proximity to prescribed fires and 7-day readmissions and at best weak evidence for a positive association between prescribed fires within 5 km and 30-day readmissions (3.2% increase per additional prescribed fire; 95% CI = -0.4%, 7.1%; Table 2).

**Table 2:** Models for prescribed fire associations with hospital visits and readmissions

Outcome	Exposure	Associated Change in Outcome (%)	95% CI (%)
Total hospital visits	Fire counts (1 km)	-5.8	-15.3, 4.2
Total hospital visits	Fire counts (2 km)	-6.0	-10.4, -1.6
Total hospital visits	Fire counts (5 km)	-2.6	-4.1, -1.1
7-day readmissions	Fire counts (1 km)	-2.1	-35.7, 49.0
7-day readmissions	Fire counts (2 km)	-7.9	-26.9, 16.3
7-day readmissions	Fire counts (5 km)	-1.8	-8.4, 5.5
30-day readmissions	Fire counts (1 km)	5.5	-15.4, 31.7
30-day readmissions	Fire counts (2 km)	-2.6	-13.6, 9.8
30-day readmissions	Fire counts (5 km)	3.2	-0.4, 7.1

*CI* Confidence interval

Associations with prescribed fires were generally unchanged under the sensitivity analyses described in the Methods. The main exception was when including observations initially identified as outliers and when restricting to patients who did not move during the study period. Under these sensitivity analyses, the direction of association with 30-day readmissions was reversed for all prescribed fire radii examined (Table S-1), but this effect was not consistently observed across outcomes. As mentioned in the Methods we also examine associations between the selected outcomes and PM_2.5_ on the day of each prescribed fire. These models only included PM_2.5_ as the exposure. For total visits the association between a 1 ug/m3 increase in PM_2.5_ was 0.5% (95% CI = 0.2, 0.8), the association with 7-day readmissions was -1.55% (95% CI = -3.0, -0.1), and the association with 30-day readmissions was -0.1% (95% CI = -0.8, 0.6). However, these associations account for only a part of the complete PM_2.5_ data available (that which intersects with the 2014–2016 study time period). For a more complete and prospective analysis of associations between PM_2.5_ and these outcomes which covers a longer time period with a larger sample size please see our previously published works on this PM_2.5_ exposure and hospital utilization among individuals with HF [30, 33].

### Smoke exposure

As described in the Methods, smoke presence data from the NOAA HMS system was employed as a metric to capture smoke exposure from larger fires, generally wildfires. The strongest associations with smoke days were observed for 30-day readmissions. There was a 0.6% (95% CI = 0.3%, 0.9%) increase in 30-day readmissions with each additional smoke day. For light smoke days, the association was 0.5% (95% CI = 0.2%, 0.9%), and for medium smoke days, the association was 6.5% (95% CI = 2.3%, 10.8%) increase in 30-day readmission. Heavy smoke days had the largest magnitude of any associations examined, with a 17.8% increase in 30-day readmissions per additional heavy smoke day (95% CI = 5.8%, 31.2%). When combining medium and heavy smoke days, we observed associations that mirrored those observed for medium smoke days alone, with a 5.4% (95% CI = 2.3%, 8.5%) increase in 30-day readmissions per additional medium or heavy smoke day (Table 3).

**Table 3:** Models of association between varying levels of smoke exposure and hospital visits and readmissions

Outcome	Exposure	Associated Change in Outcome (%)	95% CI (%)
Total hospital visits	Light smoke days	0.0	-0.2, 0.2
Total hospital visits	Medium smoke days	1.7	-0.2, 3.6
Total hospital visits	Heavy smoke days	5.4	-0.4, 11.4
Total hospital visits	Med + heavy smoke days	1.5	0.0, 3.0
Total hospital visits	Total smoke days	0.1	-0.1, 0.2
7-day readmissions	Light smoke days	0.4	-0.2, 1.5
7-day readmissions	Medium smoke days	8.1	-1.5, 18.5
7-day readmissions	Heavy smoke days	21.4	-7.0, 58.3
7-day readmissions	Med + heavy smoke days	8.5	1.5, 16.0
7-day readmissions	Total smoke days	0.9	0.2, 1.6
30-day readmissions	Light smoke days	0.5	0.2, 0.9
30-day readmissions	Medium smoke days	6.5	2.3, 10.8
30-day readmissions	Heavy smoke days	17.8	5.8, 31.2
30-day readmissions	Med + heavy smoke days	5.4	2.3, 8.5
30-day readmissions	Total smoke days	0.6	0.3, 0.9

*CI* Confidence interval

In contrast to 30-day readmissions, associations were weaker for 7-day readmissions, where the primary associations observed were with total smoke days (0.9% increase per additional smoke day; 95% CI = 0.2%, 1.6%) and medium or heavy smoke days (8.5% increase per additional medium or heavy smoke day; 95% CI = 1.5%, 16.0%). Neither medium nor heavy smoke days showed the same strength of association as the combined category (Table 3). For total hospital visits, there was evidence of a positive association for heavy smoke days (5.4% increase per additional heavy smoke day; 95% CI = -0.4%, 11.4%) and medium smoke days (1.7% increase per additional medium smoke day; 95% CI = -0.2%, 3.6%). The combined medium or heavy smoke days mirrored associations with medium smoke days for total visits; no association was seen for light smoke days or total smoke days (Table 3).

In a broad range of sensitivity analyses, associations between smoke days and 30-day readmissions were stable for medium or heavy smoke days and total smoke days (Table S-2). For other exposures, associations with 30-day readmissions were reduced for individuals who did not move, and associations with heavy smoke days were attenuated when restricted to patients less than 100 years old, causing the removal of 29 individuals listed age > 100 at the time of HF diagnosis. As associations with total hospital visits and 7-day readmissions were weaker than those observed for 30-day readmissions, we are more cautious not to over-interpret associations seen in isolated sensitivity analyses. For 7-day readmissions, associations with total smoke days and combined medium or heavy smoke days were stable in all sensitivity analyses except for attenuated associations observed with outliers retained. Associations with light smoke days and medium smoke days were strengthened when imputing smoking status, and associations with medium smoke days increased when restricted to patients less than 100 years old. For total visits, sensitivity analyses consistently showed weak to no associations. The exception was when not removing outliers, where associations were observed for heavy smoke days and medium or heavy smoke days (Table S-2). However, given the weak initial associations and the limited number of outliers, these associations are less likely to be reproducible.

## Discussion

Study findings show a negative or no association between hospital utilization and prescribed fire exposures. What associations were observed for prescribed fires were only seen for the larger radii, e.g., fires within 2 and 5 km of a primary residence, but not for those within 1 km of the primary residence, and not for all outcomes examained. The limited associations with prescribed fires, not seen for the most proximal exposures, were also in the opposite direction of what would be expected. This could be driven by factors correlated with prescribed fire occurrence outside of smoke exposure, such as ecological changes or correlated land management practices which we lacked the data to explore. In contrast, smoke exposure related to wildfires was positively associated with 30-day readmissions and, to a lesser degree, total hospital visits and 7-day readmissions. Additionally, we observed a concentration-dependent response, with heavier smoke days being more strongly associated with 30-day readmissions. Examining these associations among HF patients lends a unique but essential view, as HF patients are a particularly vulnerable subset of the community with elevated environmental health risks [31, 32, 48]. Additionally, HF prevalence is growing in the United States due to the aging of the population and the increase in HF risk factors [26, 49]. This makes studies of HF patients of particular public health importance as we seek to understand the unique environmental health risks faced by the most vulnerable community members.

Our observation of an association between smoke exposure and an increase in hospital readmissions indicates that long-term smoke exposure is a health risk to HF patients (Table 3). Though this result is expected, given the elevated environmental sensitivity of HF patients and the known links between smoke exposure and health, it is still important to quantify these health effects for HF patients and compare them with other published associations. A study of smoke exposure in the Western U.S. found that exposure to wildfire-specific PM_2.5_ (> 37 µg/m^3^) for at least two consecutive days was associated with a 7.2% increase in respiratory admissions among Medicare beneficiaries [50]. This association is comparable to the associations observed in this study for medium and medium + heavy smoke days (Table 3). The smoke exposures in our study likely come from a combination of local wildfires and long-range transport. The NOAA HMS system does not allow for tracking where the smoke originated from. However, previous studies have highlighted that smoke exposure is associated with increased readmission and mortality risks even when transported over long distances [51].

Despite the widespread usage of prescribed fires, few studies have examined prescribed fires' health impacts. One study showed that prescribed fires are associated with increased emergency room visits in asthma patients [52]. A 2021 study estimated that prescribed fire burning in Georgia increased the annual average PM_2.5_ by 0.9 µg/m^3^ in 2016. By linking modeled PM_2.5_ with established concentration–response functions, researchers showed that PM_2.5_ from prescribed fires would be projected to increase emergency department visits and mortality [25]. In our study, prescribed fire occurrences were not associated with increased hospital visits or readmissions among HF patients (Table 2). The associations were generally inverse, not observed for the most proximal prescribed fires (those within 1 km), and not robust in sensitivity analyses. While this study did not specifically examine estimates of smoke or PM_2.5_ from prescribed fires, it is the first to examine prescribed fire occurrence frequency in association with health outcomes in the community using both individual-level health and residential data and precise estimates of prescribed fire dates and location. This study is also the first to examine prescribed fire health effects in individuals with cardiovascular disease who have increased environmental health risks. Previous studies of prescribed fires using individual-level data have focused on occupational exposures experienced by firefighters, which would differ substantially from community exposures [53–55]. These studies, involving much greater exposure than the larger community would experience, showed increased inflammation and decreased lung function in association with occupational exposures to prescribed fires.

Given the advanced age (mean age equal to 70 years) and overall modest size of our study population, we did not stratify by age to investigate increased vulnerability for older individuals. Future research should address the overlapping areas of vulnerability (age, socioeconomic, clinical) which may heighten health risks related to smoke exposure. A study examining geographic and social vulnerabilities observed increased vulnerability among women and black participants [56]. However, this study did not examine clinical vulnerabilities, leaving this question unaddressed.

A limitation of this study is that it uses a cross-sectional approach which was necessitated by the limited period of exposure data. While the cross-sectional design allowed us to use all available exposure data to determine the spatial patterning of prescribed fire occurrence it comes with the limitation that some prescribed fires would have occurred after the hospital visits and hospitalizations for some individuals. This tradeoff between prospectively assessed outcomes (e.g., outcome strictly follows exposure in time) versus improved exposure assessment is often at the core of cross-sectional designs and is why cross-sectional designs cannot exclude reverse causation, making casual interpretations of results difficult. Thus, the generalizability of this study in part hinges on the spatial patterning of prescribed fires being relatively constant over time in North Carolina. Future studies with more extensive exposure and outcome data should seek to apply study designs robust to reverse causation. Despite this limitation this study does focus on a population highly sensitive to poor air quality and utilizes EHRs to capture all hospital visits, and both 7 and 30-day readmissions giving a broad picture of hospital utilization in association with prescribed fire occurrences and smoke exposure.

Another limitation of this study is that we only used a single hospital system with limited capture of individual-level socioeconomic status. The lack of individual-level socioeconomic status variables (e.g., income) is a factor faced by all EHR studies. As done here, it is typically addressed by incorporating area-level socioeconomic status indicators. Using a single hospital system might limit generalizability to a broader population. However, this and previous studies based on this patient population have shown concordance with patient populations across the U.S., suggesting the results may generalize beyond a single hospital system [30, 32, 33].

Another limitation is that we did not have direct measures of smoke emitted by prescribed fires. While the pollution concentration increases with proximity to prescribed fires, the occurrence of prescribed fires is only a proxy for pollution exposure and potential longer-term ecological benefits. Future studies should incorporate more direct measurements of smoke exposure, possibly using mobile monitoring at both residences and near the prescribed fires to accurately assess the emitted pollutants. Nevertheless, we believe that the occurrence of prescribed fires nearby to the primary residence is a critical exposure metric with the potential to inform both community members and policymakers in charge of prescribed fire programs on the potential health impacts of this land management tool.

## Conclusions

In conclusion, we observed substantial health effects from smoke exposure associated with wildfires among HF patients. Similar associations were not observed for local prescribed fire occurrences, suggesting that prescribed fires, as implemented during the study time frame, may not contribute to health effects among HF patients in the same manner as wildfire smoke exposure. Future studies should continue to explore smoke-related health risks in vulnerable populations while examining prescribed fire programs for evidence of health effects.

### Supplementary Information

**Additional file 1:** **Figure S-1.** Histogram of 7-day readmissions. This histogram was used to remove the outlying observations for number of 7-day readmissions.

**Additional file 2:** **Figure S-2.** Histogram of 30-day readmissions. This histogram was used to remove the outlying observations for the number of 30-day readmissions.

**Additional file 3:** **Table S-1.** Sensitivity analyses for associations between prescribed fires and hospital visits and readmissions.

**Additional file 4:** **Table S-2.** Sensitivity analyses for associations between smoke exposure and hospital visits and readmissions.

**Additional file 5. **

**Additional file 6. **

## Data Availability

This material is based on work supported by the National Science Foundation under Grant No. 1751601. Publicly available data used in this study can be found here: HMS smoke density data, US Census Bureau Data, Prescribed fires and emissions modeling platform. Data containing protected health information is not publicly shareable and can only be obtained upon reasonable request with appropriate Institutional Review Board application approval.

## Abbreviations

CI: Confidence Interval
EHR: Electronic Health Record
EPA CARES: Environmental Protection Agency Clinical and Archived Records Research for Environmental Studies
GOES: Geostationary Operational Environmental Satellites
HF: Heart Failure
ICD-9: The International Classification of Diseases, Ninth Revision
ICD-10: The International Classification of Diseases, Tenth Revision
IQR: Interquartile Range
IRB: Institutional Review Board
MTBS: Monitoring Trends in Burn Severity
NOAA HMS: National Oceanic and Atmospheric Administration’s Hazard Mapping System
PM_2.5_: Fine Particulate Matter
R: R statistical computing software
SD: Standard Deviation
SMARTFIRE2: Satellite Mapping Automated Reanalysis Tool for Fire Incident Reconciliation version 2
UNCHCS: University of North Carolina Healthcare System
ZCTA: Zip Code Tabulation Area
