# Supplementary figures and images for: Prescribed fires, smoke exposure, and hospital utilization among heart failure patients
Source: Environ Health. 2023 Dec 13;22:86. doi: 10.1186/s12940-023-01032-4 (PMC10717133; doi:10.1186/s12940-023-01032-4)

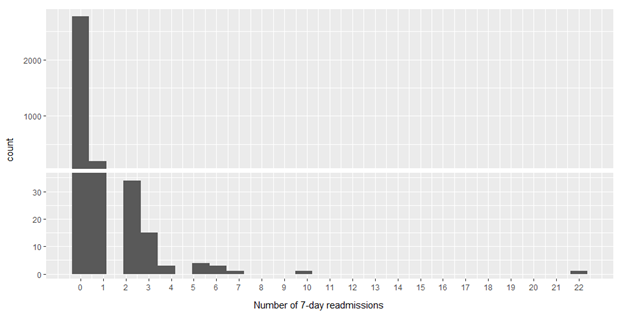

Supplement: Supplementary file 1 — Additional file 1: Figure S-1. Histogram of 7-day readmissions. This histogram was used to remove the outlying observations for number of 7-day readmissions. [file 12940_2023_1032_MOESM1_ESM.png]

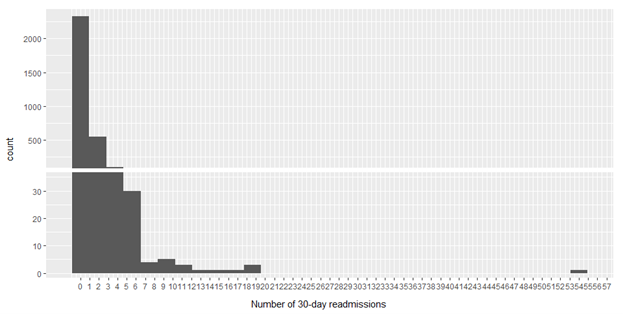

Supplement: Supplementary file 2 — Additional file 2: Figure S-2. Histogram of 30-day readmissions. This histogram was used to remove the outlying observations for the number of 30-day readmissions. [file 12940_2023_1032_MOESM2_ESM.png]
